# Divergence in Plant Traits and Increased Modularity Underlie Repeated Transitions Between Low and High Elevations in the Andean Genus *Leucheria*

**DOI:** 10.3389/fpls.2020.00714

**Published:** 2020-06-04

**Authors:** Fernanda Pérez, Nicolás Lavandero, Carmen Gloria Ossa, Luis Felipe Hinojosa, Paola Jara-Arancio, Mary T. Kalin Arroyo

**Affiliations:** ^1^Departamento de Ecología, Facultad de Ciencias Biológicas, Pontificia Universidad Católica de Chile, Santiago, Chile; ^2^Instituto de Ecología y Biodiversidad, Santiago, Chile; ^3^Instituto de Biología, Facultad de Ciencias, Universidad de Valparaíso, Santiago, Chile; ^4^Departamento de Ciencias Ecológicas, Facultad de Ciencias, Universidad de Chile, Santiago, Chile; ^5^Departamento de Ciencias Biológicas y Departamento de Ecología y Biodiversidad, Facultad de Ciencias para la Vida, Universidad Andrés Bello, Santiago, Chile

**Keywords:** climatic niche lability, coordinated evolution, evolutionary rates, plant traits, high Andes, modularity, phenotypic integration

## Abstract

Understanding why some plant lineages move from one climatic region to another is a mayor goal of evolutionary biology. In the southern Andes plant lineages that have migrated along mountain ranges tracking cold-humid climates coexist with lineages that have shifted repeatedly between warm-arid at low elevations and cold habitats at high elevations. Transitions between habitats might be facilitated by the acquisition of common traits favoring a resource-conservative strategy that copes with drought resulting from either low precipitation or extreme cold. Alternatively, transitions might be accompanied by phenotypic divergence and accelerated evolution of plant traits, which in turn may depend on the level of coordination among them. Reduced integration and evolution of traits in modules are expected to increase evolutionary rates of traits, allowing diversification in contrasting climates. To examine these hypotheses, we conducted a comparative study in the herbaceous genus *Leucheria.* We reconstructed ancestral habitat states using Maximum Likelihood and a previously published phylogeny. We performed a Phylogenetic Principal Components Analysis on traits, and then we tested the relationship between PC axes, habitat and climate using Phylogenetic Generalized Least Squares (PGLS). Finally, we compared the evolutionary rates of traits, and the levels of modularity among the three main Clades of *Leucheria*. Our results suggest that the genus originated at high elevations and later repeatedly colonized arid-semiarid shrublands and humid-forest at lower elevations. PGLS analysis suggested that transitions between habitats were accompanied by shifts in plant strategies: cold habitats at high elevations favored the evolution of traits related to a conservative-resource strategy (thicker and dissected leaves, with high mass per area, and high biomass allocation to roots), whereas warm-arid habitats at lower elevations favored traits related to an acquisitive-resource strategy. As expected, we detected higher levels of modularity in the clades that switched repeatedly between habitats, but higher modularity was not associated with accelerated rates of trait evolution.

## Introduction

Understanding why some plant lineages transit between contrasting climatic regions whereas others retain their ancestral climatic niche is a major goal of evolutionary biology (Losos, 2008; Crisp et al., 2009; Wiens et al., 2010; Edwards and Donoghue, 2013). Shifts between contrasting climatic regions may be accompanied by shifts in traits that allow species to cope with new environmental conditions or alternatively may be facilitated by the preexistence of “enabler” traits (Harvey and Pagel, 1991; Edwards and Donoghue, 2013; Ogburn and Edwards, 2015). Zanne et al. (2014), for example, showed that some traits that allow species to survive in cold conditions were acquired before lineages forayed into the cold, whereas others evolved in response to freezing temperatures. These observations suggest that some traits are more labile than others, and as claimed by Stebbins (1950) evolution will occur along the lines of least resistance.

The evolutionary lability of a trait may depend on the intensity of natural selection and its genetic variation, as well as on genetic interactions with other traits (Lande, 1976; Pigliucci, 2003). Genetic interactions among traits are expected to constrain adaptive evolution and bias phenotypic divergence among species toward the genetic lines of least resistance, defined as the combination of traits with maximum genetic variance within a population (Schluter, 1996). As predicted by this hypothesis covariation patterns of phenotypic traits among species tend to be aligned with genetic correlations within populations (Schluter, 1996, 2000; McGuigan et al., 2005). Although the signature of genetic constraints is expected to decline over time (Schluter, 1996), some studies have shown that this can persist over longer evolutionary scales (Houle et al., 2017; McGlothlin et al., 2018). The tendency of traits to vary coordinately across con-specific individuals or phylogeny is known as phenotypic integration (Klingenberg, 2014) and can play an important role in constraining and canalizing morphological evolution (Lewontin, 1978; Wagner et al., 2007; Klingenberg, 2008). In contrast, evolutionary lability can be facilitated by the parceling of phenotypes into “functional modules.” The latter are, clusters of functionally-linked traits that tend to evolve in a coordinated fashion, but independent of other suites of traits that are associated with other functions (Wagner and Altenberg, 1996). Given that functional modules are independent from surrounding traits in their fitness effects, some modules can diverge more rapidly than others and generate more disparity (Evans et al., 2017; Larouche et al., 2018). Accordingly, evolution of traits in functional modules might enable accelerated rates of trait evolution (Breuker et al., 2006; Claverie and Patek, 2013), allowing diversification in contrasting climatic regions.

Mountainous vegetation above tree line (high elevation habitats) harbors a high diversity of species with different biogeographic origins (Simpson, 1983; Arroyo and Cavieres, 2013; Antonelli, 2015). Important sources of mountainous floras are temperate and boreal lineages that migrated along mountain chains (or by long distance dispersion) tracking cold conditions; these elements coexist with lowland elements that evolved under warmer climates but managed to invade cool conditions. Major challenges for colonization of high-elevation environments result from the extreme and variable weather conditions. With increasing elevation, temperature drops (an average of 5.5°C per kilometer of altitude) and clear-sky solar radiation increases (Korner, 2007). In mediterranean-climate mountainous areas, these trends are usually accompanied by an increase in precipitation (Korner, 2007), reduction in the length of the growing season (Kudo, 1991), and reduction in soil fertility (Cavieres et al., 2000). How high-elevation plants deal with cold, short growing seasons and low resource availability are long-standing questions in alpine ecology (Korner, 2003). However, few studies have used a comparative approach, and therefore it is not well known whether traits that allow species to survive in high elevation habitats evolved before or in response to freezing temperatures, and whether these traits evolved in a coordinated fashion.

The coordinated evolution of traits might result from allocation to one function vs. another (Reich, 2014). Indeed, despite the high diversity of traits observed in vascular plants, only some trait combinations are viable and successful, indicating strong coordination and trade-offs between plant traits (Díaz et al., 2016). One of the more remarkable trade-offs is produced by the balance between rapid acquisition and efficient resource conservation, reflected in the worldwide leaf economics spectrum (LES; Reich et al., 1997; Wright et al., 2004). The LES was originally defined by six leaf traits, but now has been extended to the whole plant in a single fast-slow plant economic spectrum (Tjoelker et al., 2005; Reich, 2014). Traits related to a resource-conservative strategy (such as long-lived, thick leaves with high leaf mass per area, reduced N content and low photosynthetic rate, and high biomass assignation to roots) are expected to occur in species with drought stress and low resource availability, including high elevation (Korner, 2003; Read et al., 2014) and arid habitats (Knight and Ackerly, 2003).

The flora of the southern Andes that occurs above the treeline (high Andean plants) exhibits strong floristic relationships with the flora of arid and semiarid regions of the Atacama Desert and the mediterranean-climate shrublands of Central Chile (Luebert, 2011). These relationships are based on the strong effect that the Andes uplift had at the onset of arid and semiarid conditions in northern and central Chile, which were also reinforced by the establishment of the Humboldt Current (Hinojosa and Villagran, 1997; Hinojosa, 2005). Phylogenetic studies in various genera indicate that frequent shifts between these regions took place in both directions since the Miocene (Luebert and Weigend, 2014). One genus that has seen such shifts is *Leucheria* (Tribe Nassauvieae Cass, subfamily Mutisioideae Cass., family Asteraceae), a South American genus composed of 47 herbaceous species distributed from 11 to 54°S and from sea level to 5000 m. elevation in a wide range of habitats, mostly in Chile and Argentina (Crisci, 1976; Jara-Arancio et al., 2017). Transitions between low and high elevation habitats in this group might be facilitated by the acquisition of “conservative” traits that cope with drought and limiting nutrients availability that result either from low precipitation or extreme cold. Alternatively, some trade-offs between drought and cold resistance can occur, promoting the evolution of specific adaptations to each environment.

In this study we combined phylogenetic inference, with elevation and climate data to reconstruct the evolutionary sequence of habitat shifts within *Leucheria*. We also measured several traits related to leaf morphology (area, mass per area, thickness, dissection, aspect ratio) and life form (root-to-shoot biomass and plant height) to explore: (i) whether transitions between cold high-elevation habitats and arid-semiarid shrublands at lower elevations were facilitated by the acquisition of common “conservative” traits, or to the contrary were accompanied by shifts in plant strategies; and (ii) whether leaf and life from traits evolved coordinately, or parceled into modules. We also estimated the level of modularity and the evolutionary rates in the three main clades of *Leucheria*. Based on these analyses we tested (iii) whether clades that transit between contrasting climatic regions have increased modularity and exhibit higher rates of trait evolution.

## Materials and Methods

### Plant Material

We collected plant material of 34 species from sites (one site per species) located between 29 and 52°S in Chile over and altitudinal range from 0 to 3500 m. One sun-exposed leaf per individual from 10 individuals per species was collected. Leaves were scanned and dried in an oven at 60°C for 2 days to obtain estimates of leaf area (LA) and leaf mass per area (LMA = dry mass/fresh leaf area; Cornelissen et al., 2003). The degree of leaf dissection (LD; perimeter/2${}^{*}\,\pi \,\sqrt{L\,A}$ ratio) and the aspect ratio of leaves (AR, length/width ratio) were also estimated using ImageJ (Schneider et al., 2012). The leaf thickness (LT) was measured halfway along the length of the leaf and halfway between the midrib and leaf edge using an analog thickness gauge (±0.01 mm; Fowler, Japan). We also collected 10 entire plants of each species in order to estimate the plant height and the ratio of the belowground to aboveground biomass (root/shoot ratio, RSR).

### Evolutionary Shifts in Habitats and Plant Traits

**FIGURE 1:**
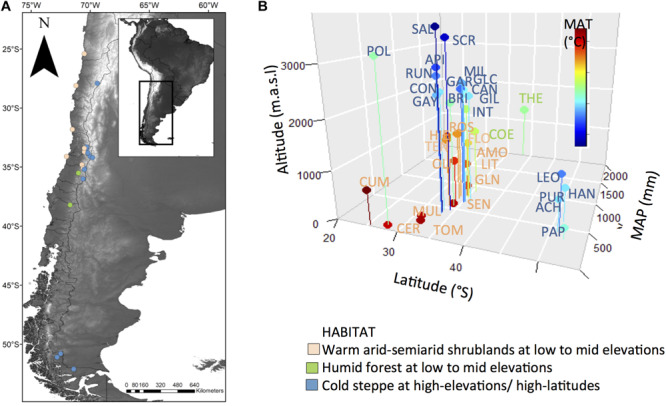
**(A)** Map of Chile showing locations of studied sites colored according to habitat. **(B)** Plot of altitude, latitude and mean annual precipitation (MAP) of studied localities of each species. Dots were colored according to the mean annual temperature (MAT) of the studied sites. Species abbreviations were colored according to habitat (see species name in Supplementary Appendix 2).

To reconstruct the evolutionary sequence of shifts in habitat within *Leucheria* we used the majority rule consensus tree recovered from the Bayesian analyses of three DNA regions (Jara-Arancio et al., 2017). The unstudied species were pruned from the original phylogeny. The consensus tree was made ultrametric and the single polytomy detected was resolved by randomly assigning a branch length equal to 0.0001. The ancestral habitat was reconstructed using the re-rooting method of Yang et al. (1995) implemented in the R package phytools (Revell, 2012) under an ER model (equal rates for all permitted transitions). Previously, we performed a likelihood ratio test to compare the goodness of fit of this model with a SYM model (symmetric backward and forward rates for all permitted transitions); the first was selected because the SYM model did not led to a significant increase in likelihood (Log-likelihood: model ER = −29.7, model SYM = −27.5; $\chi_{5}^{2}$ = 4.4, *P* = ns). We coded habitat as: WA, warm arid-semiarid shrublands at low to mid elevations of coastal desert and the Mediterranean-climate region of Central Chile; HF, humid forest; CS, cold steppe at high-elevations (above treeline) or high-latitudes in Patagonia (Figure 1). Altitudinal treeline is around 2200 m at 33°S and declines with latitude. Some species grow in more than one habitat, and given that the method cannot incorporate polymorphic data at the tips, we assign each of these species to the habitat in which it was more common (>50% of their distribution).

To explore whether transitions between habitats were accompanied by shifts in plant traits, we performed a phylogenetic principal components analysis (PCA) (Revell, 2009) on standardized morphological traits. Then, we performed Ordinary Least Squares (OLS) and Phylogenetic Generalized Least Squares (PGLS) analyses using PC axes as dependent variables, and habitat or variables describing species climatic niche (see below) as predictors. PGLS were estimated using Pagel’s lambda (λ) transformation in the R-package Caper 3.1.3 (Orme, 2013). To compare the OLS and PGLS models we used the Akaike information criterion (AIC). We also estimated phylogenetic signal for each trait separately (λ) (Pagel, 1999) using a maximum likelihood (ML) approach implemented in the R-package geiger (Harmon et al., 2008). A value of λ = 0 indicates no phylogenetic signal in a trait, while a value of λ = 1 indicates that the trait evolved according to Brownian motion in the phylogeny (Pagel, 1999). The realized climatic niche of each species was estimated using the maximum entropy approach with MAXENT (Phillips et al., 2006), and the 19 bioclimatic and topographic variables contained in the WorldClim climate data base (Hijmans et al., 2005) at one km (30 s) resolution. Information on the distribution of each species was compiled from herbarium records, the literature and new field-collected data (Supplementary Appendix 1). Background points were randomly chosen within the area enclosed by a minimum convex polygon comprising all records of the species. Occurrence data were partitioned 100 times into training and test data (80 and 20%, respectively) for model evaluation using the operating characteristic curve (AUC). During these runs, the relative contribution of each variable to the final model was automatically determined by MAXENT. Probability (suitability) distributions derived from MAXENT were used to obtain predicted niche occupancy profiles of each species with respect to mean annual temperature (MAT) and mean annual precipitation (MAP). We estimated a weighted mean of each climatic variable for each species to be used in comparative analyses (hereafter, w-minT, w-MAT, w-MAP). All analyses were conducted in the R-package phyloclim (Heibl, 2011).

### Modularity and Evolutionary Rates

To explore whether leaf and life form traits evolved coordinately or parceled into modules, we used the covariance ratio coefficient (CR; Adams, 2016) estimated from the phylogenetically independent contrast of standardized log-transformed traits. CR represents the ratio of the covariance between groups relative to the covariance within groups. Analyses were performed on all species and on each of the three main clades of *Leucheria* separately using the function modulary.test of the R-package geomorph (Adams et al., 2019). We also estimated the rate of evolution for each trait from the variance of standardized contrasts (ci) on log-transformed data, as Σci^2^/(n-1), where n is the number of taxa. F tests were performed to test for differences in rates between clades (Ackerly, 2009) and among traits. These analyses were performed in the R- package ape (Paradis et al., 2004).

## Results

### Evolutionary Shifts in Habitat and Plant Strategies

**FIGURE 2:**
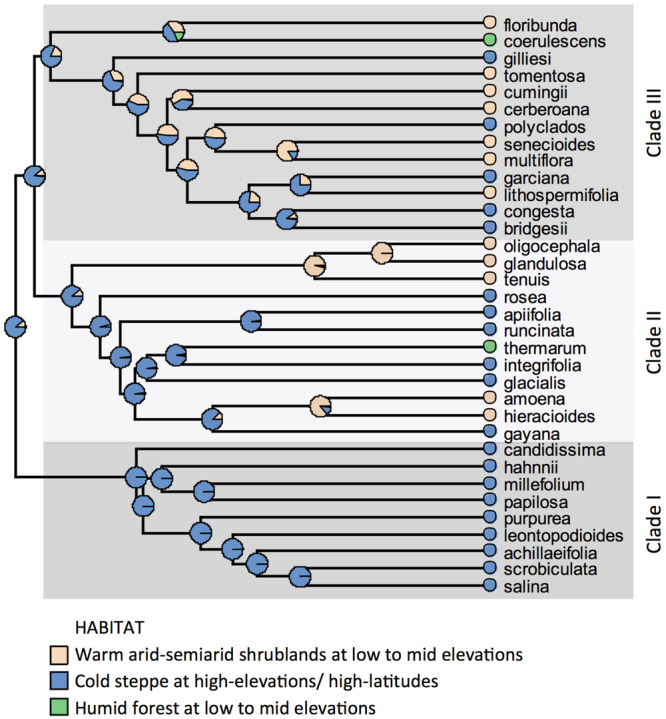
Maximum likelihood ancestral state reconstruction of habitat under a symmetric backward/forward rate model on the majority rule consensus tree of *Leucheria* recovered from Bayesian analyses of three DNA regions.

Three main clades can be recognized in the phylogeny of *Leucheria.* All species from Clade I grow in cold (wMAT: range = 1.9–6.7°C) and semiarid to sub-humid conditions (wMAP: range = 366–738 mm). Species from Clades II and III grow under a wider range of temperatures (wMAT: range = 3.7–15.6°C) and precipitation (wMAP: range = 52–1200 mm). Maximum likelihood ancestral state reconstruction of habitat suggested that the genus originated in cold habitats at high elevations and later colonized arid-semiarid shrublands and humid forest at lower elevations (Figure 2). Five switches from high to low elevations and two switches in the opposite direction were detected in Clades II and III.

**FIGURE 3:**
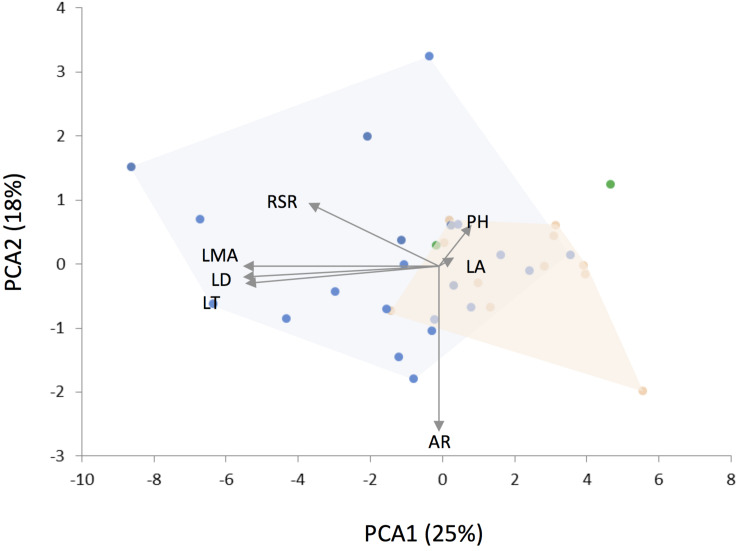
Results of phylogenetic principal component analysis (PCA) for 34 *Leucheria* species colored according to their habitat (see Figure 1). Arrows were scaled to represent the loadings of plant traits: RSR, root/shoot ratio; PH, plant height; LMA, leaf mass per area; LD, leaf dissection; LT, leaf thickness; LA, leaf area; AR, leaf aspect ratio.

The phylogenetic PCA on plant traits segregated *Leucheria* species according to their habitat (Figure 3). The first component, which explained 25% of the variance, was negatively correlated with LMA, leaf dissection, leaf thickness and RSR. Thus, low values for PCA1 reflects stronger ability to conserve resources, whereas high values reflect stronger ability to capture and use resources. The second PCA component (18% of variance) was negatively correlated with leaf aspect ratio (i.e., leaves of species with higher values of PCAII were more elongated), whereas the third PCA component (15% of variance) was positively correlated with plant height and leaf area. PGLS analyses revealed a significant effect of habitat on PCA1 [*F*_(2,27)_ = 7.94, *p* = 0.001, λ = 0.62; Table 1]: species that grow in cold habitats at high elevations or latitudes have lower values for this component, whereas species that grow in warm arid-semiarid habitats or in humid forest show the highest values. A positive and significant effect of MAT [*F*_(2,27)_ = 3.85, *p* = 0.01, λ = 1] on PCA1 was also detected. In other words, species from colder habitats showed traits related to a conservative-resource strategy. No significant effect of MAT, MAP on PCA2 or PCA3 was detected. Models including the maximum likelihood estimate of λ fit better than the model with lambda fixed at zero as indicated by lower AICs values, indicating significant phylogenetic signal in the data. Maximum likelihood estimations of λ did not differ from 1.00 for climatic niche variables and for all traits, except for aspect ratio.

**TABLE 1:** Statistics for PGLS analyses on PCA axes.

	**PCA I**	**PCAII**
Habitat	*F*_(2,27)_ = 7.94, *p* = 0.001, *R*^2^ = 0.37, λ = 0.62	*F*_(2,27)_ = 2.67, *p* = 0.09, *R*^2^ = 0.17, λ = 0.07
Int (CS)	*t* = −2.10, *p* = 0.04	*t* = −1.28, *p* = 0.21
HF	*t* = 3.26, *p* = 0.003	*t* = 1.76, *p* = 0.09
WA	*t* = 3.03, *p* = 0.005	*t* = 2.02, *p* = 0.05
Climate	*F*_(2,27)_ = 3.09, *p* = 0.05, *R*^2^ = 0.22, λ = 0.60	*F*_(2,27)_ = 1.66, *p* = 0.21, *R*^2^ = 0.11, λ = 0.26
MAT	*t* = 2.77, *p* = 0.01	*t* = 1.76, *p* = 0.09
MAP	*t* = 0.24, *p* = 0.81	*t* = 0.58, *p* = 0.57

*Habitat: WA, Warm arid-semiarid shrublands (low to mid elevations); HF, Humid forest (low to mid elevations); CS, Cold steppe (high-elevations/latitudes). Climatic variables estimated from predicted niche occupancy: wMAT, weighted mean of mean annual temperature; wMAP, weighted mean of annual precipitation. Pagel′s λ was estimated by maximum likelihood.*

In agreement with PCA results, modularity analyses for all species showed that life form and leaf traits did not evolve independently (CR = 2.34, *p* = 0.64). The same tendency was detected in Clade I (CR = 1.98, *p* = 0.49), but not in Clade II (CR = 0.64, *p* = 0.02) or Clade III (CR = 1.07, *p* = 0.07). In these clades CR values between leaf and life form traits were lower than expected by chance, indicating some degree of modularity. Contrary to the expected, we did not detect significant differences in evolutionary rates among leaf and plant traits when all *Leucheri*a species were analyzed together (Table 2). Likewise, reduced modularity in Clade I was not associated with an increment in evolutionary rates of all functional traits; LMA and leaf thickness evolved faster in Clade I than in the other two clades, whereas root-to-shoot ratio evolved faster in Clades II and III.

**TABLE 2:** Phylogenetic signal and evolutionary rates of traits and climatic niche.

	**Phylogenetical signal**			**Evolutionary rates (β)**			
	
**Trait**	**λ**	**AIC λ = 0**	**AIC λ = 1**	**Total**	**Clade I**	**Clade II**	**Clade III**
PH	1.00	343	327*	5.7^a^	4.48^A^	5.81^A^	2.10^A^
LA	1.00	225	204*	5.0^a^	4.66^A^	3.96^A^	9.25^B^
LMA	0.90	364	352*	6.5^a^	9.95^B^	3.25^A^	5.72^AB^
LT	0.85	−71	−79*	7.3^a^	5.46^A^	3.30^A^	13.2^B^
LD	1.00	34	21*	6.1^a^	9.95^B^	3.25^A^	5.71^AB^
AR	0.00	135*	142	10.5^a^	13.0^B^	19.7^B^	2.31^A^
RSR	0.94	87	82*	7.6^a^	0.86^A^	11.3^B^	8.87^B^

*AIC, Akaike information criterion for estimated λ (AIC k) and for testing models with λ = 0 (white noise) and λ = 1 (BM). Asterisks indicate the selected model with the lowest AIC. Evolutionary rates were estimated for all species and for each clade separately. Significant differences in rates among traits are indicated with lowercase letters in column “Total” and significant differences among clades are indicated with capital letters (tested for each trait separately). Plant traits: RSR, root/shoot ratio; PH, plant height; LMA, leaf mass per area; LD, leaf dissection; LT, leaf thickness; LA, leaf area; AR, leaf aspect ratio (Figure 2).*

## Discussion

Our results suggest that the *Leucheria* clade originated in cold habitats at high elevations/latitudes and later colonized arid-semiarid shrublands and humid-forest at lower elevations. Switches from low to high elevations also occurred, suggesting that downward and upward migrations took place several times in the genus. Switches between low and high elevation also occurred in the sister group to *Leucheria*, which is comprised by *Marticorenia* and *Moscharia* (Jara-Arancio et al., 2017): the first genus has a single species that grows at high elevations, whereas the second genus is composed of two annual species that grow at low elevations. There is a good possibility that the two low elevation annual species of *Moscharia* are derived from higher elevation perennial *Moscharia*, but this needs to be verified. Similar diversification patterns have been documented for other South American herbaceous groups, such as the *Chaetanthera*-*Oriastrum* lineage, which had a high Andean origin (Hershkovitz et al., 2006), and *Oxalis* (Heibl and Renner, 2012), which entered high Andes from arid lowlands at least six times. Shifts between high Andes and Patagonian steppe are also common (Heibl and Renner, 2012), but in general most genera show diversification patterns consistent with a south-to-north migration (e.g., *Azorella*, Nicolas and Plunkett, 2012; *Chuquiraga*, Gruenstaeudl et al., 2009). In these cases, the Andes appear to have acted as a corridor, facilitating the dispersion of lineages that track cold and semi-humid conditions (Luebert and Weigend, 2014).

Transitions between cold high-elevation habitats and arid-semiarid shrublands at lower elevations were not facilitated by the acquisition of common traits to deal with drought. Instead, our results show that transitions between habitats were accompanied by shifts in plant strategies: cold climates of the Patagonian steppe and high Andes favored traits on the “slow end” of the plant economic spectrum, whereas arid-semiarid and humid climates favored traits related to the “fast end.” Thicker leaves, with high mass, and high biomass allocation to roots are common in alpine environments (Korner, 2003), but traits related to a resource-acquisitive strategy are expected to occur in nutrient-rich climatic conditions. Nevertheless, these expectations are based on studies that combine species with different life forms (Mason and Donovan, 2015). Rapid use of limited resources can be favored in herbs that grow in hot and dry habitats as part of a fast development strategy associated with short life cycles and autonomous selfing, which together allow completion of the life cycle before the onset of unfavorable conditions (Aarssen, 2000; Snell and Aarssen, 2005; Kooyers, 2015). For example, Mason and Donovan (2015) reported that herbaceous *Helianthus* species from drier environments adopt a more-acquisitive-resource strategy than species from cooler and rich environments. Likewise, several comparative studies show that shifts from perennial to annual life histories are frequent in hot and dry conditions (Evans et al., 2005; Friedman and Rubin, 2015). For example, in the genus *Centaurium* four of three transitions from perennial to annual habit occurred during periods of aridization (Jímenez-Lobato et al., 2019). The evolution of a rapid developmental strategy is also associated with the acquisition of autonomous selfing and reduced allocation to floral structures (Aarssen, 2000; Snell and Aarssen, 2005). In agreement with this pattern, we observed that annual species of *Leucheria* tend to have smaller capitula (flower heads) than perennial species, suggesting that annuals have the capacity for autonomous selfing. Furthermore, the two perennial species with known reproductive systems are strongly adapted for outcrossing (Arroyo and Squeo, 1990). Shifts from annual to perennial life form are frequent in high elevation habitats (Drummond et al., 2012; Hughes and Atchison, 2015). High inter-annual climate variability, short growing seasons and repeated frosts can inhibit seedling establishment and reduce the probability of completing a life cycle in one season (Bliss, 1971).

PCA analyses showed that plant traits in *Leucheria* tend to evolve in modules. Interestingly, the level of integration among modules was lower in Clades II and III than in Clade I, supporting the hypothesis that reduced integration (or higher modularity) allows diversification in contrasting climates. One expected consequence of modularity is that some modules can diverge more rapidly than others and generate more disparity (Evans et al., 2017; Larouche et al., 2018). Modularity is also expected to favor higher evolutionary rates (Claverie and Patek, 2013; Collar et al., 2014). Contrary to these hypotheses, we did not detect significant differences in evolutionary rates among plant traits belonging to different modules when all *Leucheria* species were analyzed together. Increased phenotypic integration in Clade I was not associated with a reduction in evolutionary rates of all functional traits. Other macroevolutionary studies have also failed to find a correlation between integration and evolutionary rates (Goswami et al., 2014). As discussed by Goswami et al. (2014), high integration among traits can constrain or promote trait evolution depending on whether the major axis of morphological covariation does or does not align with the direction of selection on those traits. The major axis of phenotypic variation among species is usually associated with the maximum genetic variance within populations (g_*max*_; Schluter, 1996), and accordingly can be interpreted as the genetic line of least resistance. In the case of *Leucheria*, the PCA1 is associated with the fast-slow economic spectrum and correlates with climate, suggesting that the patterns of phenotypic integration and underlying genetic correlations are shaped by natural selection. However, it is important highlight that one limitation of our study is that we examined the patterns of covariation across phylogeny using a single species per locality. Further studies at smaller taxonomic scales are necessary to explore whether the patterns of phenotypic integration and the relationship between traits and climate remain stable at lower evolutionary scales.

We found evidence of phylogenetic signal in most plant traits and climatic niche variables. Strong phylogenetic signal is usually interpreted as a sign of phylogenetic constraints and low evolutionary lability, but as shown by Revell et al. (2008) different evolutionary processes can produce similar phylogenetic signals. In the case of *Leucheria*, PGLS analyses revealed a significant effect of habitat on plant traits, suggesting that high phylogenetic signal does not reflect phylogenetic inertia, but rather a tendency for related species to inhabit similar conditions. For example, all species of Clade I grow in cold steppe at high latitudes or altitudes.

Our results showed that root-to-shoot ratio, leaf thickness, LMA and leaf dissection tend to evolve in concert, and separately from plant height, leaf area and aspect ratio. It is well known that variation in LMA among species is driven by variation in leaf thickness and leaf density (Vile et al., 2005), although the relative importance that has been assigned to each component varies among studies (Vile et al., 2005; Poorter et al., 2009). However, the relationship between LMA and thickness with leaf shape has been less studied. Leaf thickness limits mesophyll conductance to CO_2_ diffusion (Flexas et al., 2008), whereas leaf dissection reduces the depth of the boundary layer, which in turn increases CO_2_ diffusion into leaves (Kidner and Umbreen, 2010). Accordingly, higher dissection in thicker leaves might evolve as an adaptation that compensates the effect of thickness on CO_2_ diffusion. Alternatively, the three leaf traits might respond to similar environmental conditions. For example, thicker leaves with high mass increase resistance to drought and to mechanical tension produced by wind. Leaf dissection increases hydraulic conductance (Sack and Hoolbrook, 2006) and avoids drag produced by wind (Vogel, 2009). Our results also provide evidence for correlated evolution between these traits and biomass allocation to roots, supporting the hypothesis that plant adaptation requires the integration of traits from different organs (Reich, 2014). The species that reach the highest elevation in their respective habitats (*L. salina* and *L. scrobiculata* in Central Chile, and *L. leontopodioides* in Patagonia) show massive investment in belowground stems or tap roots, which has also been observed in other species of high elevation habitats (Korner, 2003). These structures might store enough resources to produce the “expensive” thick leaves in the short time that the growing season lasts in these habitats, or alternatively, might provide more resistance to mechanical tensions produced by freeze-thaw cycles (Korner, 2003).

Overall, our results show that functional traits evolved in modules, at similar rates and in concert with habitat and climate. As expected, we detected higher levels of modularity in the clades that switches repeatedly between high and low elevations, supporting the hypothesis that reduced integration (or higher modularity) allows diversification in contrasting climates. However further research on other taxa is needed to explore whether high climatic niche lability is usually associated with changes in trait correlations in addition to shifts in trait values.

## Data Availability Statement

All datasets generated for this study are included in the article/Supplementary Material.

## Author Contributions

FP, MA, and LH formulated the idea. NL, CO, and FP conducted fieldwork. LH, PJ-A, and FP performed statistical analyses. FP wrote the manuscript.

## Conflict of Interest

The authors declare that the research was conducted in the absence of any commercial or financial relationships that could be construed as a potential conflict of interest.

The reviewer IL declared past co-authorship with several of the authors FP, CO, and MA to the handling Editor.

## References

[B1] Aarssen L. W. (2000). Why are most selfers annuals? A new hypothesis for the fitness benefit of selfing. *Oikos* 89 606–612. 10.1034/j.1600-0706.2000.890321.x 15707481

[B2] Ackerly D. (2009). Conservatism and diversification of plant functional traits: evolutionary rates versus phylogenetic signal. *Proc. Natl. Acad. Sci. U.S.A.* 106 19699–19706. 10.1073/pnas.0901635106 19843698 PMC2780941

[B3] Adams D. C. (2016). Evaluating modularity in morphometric data: challenges with the RV coefficient and a new test measure. *Methods Ecol. Evol.* 7 565–572. 10.1111/2041-210X.12511

[B4] Adams D. C. Collyer M. Kaliontzopoulou A. (2019). *Geomorph: Software for Geometric Morphometric Analyses. R package version 3.1.0.* Available online at: https://cran.r-project.org/package=geomorph

[B5] Antonelli A. (2015). Biodiversity: multiple origins of mountain life. *Nature* 524 300–301. 10.1038/nature14645 26266978

[B6] Arroyo M. T. K. Cavieres L. (2013). “High-elevation andean ecosystems,” in *Encyclopedia of Biodiversity*, ed. Levin S. A. (New York, NY: Springer), 96–110. 10.1016/B978-0-12-384719-5.00428-7

[B7] Arroyo M. T. K. Squeo F. A. (1990). “Relationship between plant breeding systems and pollination,” in *Biological Approaches and Evolutionary Trends in Plants*, ed. Kawano S. (London: Academic Press), 205–227.

[B8] Bliss L. C. (1971). Arctic and alpine plant life cycles. *Annu. Rev. Ecol. Syst.* 2 405–438. 10.1146/annurev.es.02.110171.002201

[B9] Breuker C. J. Debat V. Klingenberg C. P. (2006). Functional evo–devo. *Trends Ecol. Evol.* 21 488–492. 10.1016/j.tree.2006.06.003 16806575

[B10] Cavieres L. A. Peñaloza A. Arroyo M. T. K. (2000). Pisos altitudinales de vegetación en los Andes de Chile central (33°S). *Rev. Chil. Hist. Nat.* 73 331–344. 10.4067/S0716-078X2000000200008 27315006

[B11] Claverie T. Patek S. N. (2013). Modularity and rates of evolutionary change in a power-amplified prey capture system. *Evolution* 67 3191–3207. 10.1111/evo.12185 24152002

[B12] Collar D. C. Wainwright P. C. Alfaro M. E. Revell L. J. Mehta R. S. (2014). Biting disrupts integration to spur skull evolution in eels. *Nat. Commun.* 5:5505. 10.1038/ncomms6505 25399532

[B13] Cornelissen J. H. C. Lavorel S. Garnier E. Díaz S. Buchmann N. Gurvich D. E. Reich P. B. (2003). Handbook of protocols for standardised and easy measurement of plant functional traits worldwide. *Aus. J. Bot.* 51, 335–380. 10.1071/BT02124

[B14] Crisci J. V. (1976). Revisión del género *Leucheria* (Compositae: Mutisieae). *Darwiniana* 20 9–126.

[B15] Crisp M. D. Arroyo M. T. Cook L. G. Gandolfo M. A. Jordan G. J. McGlone M. S. (2009). Phylogenetic biome conservatism on a global scale. *Nature* 458 754–756. 10.1038/nature07764 19219025

[B16] Díaz S. Kattge J. Cornelissen J. H. C. Wright I. J. Lavorel S. (2016). The global spectrum of plant form and function. *Nature* 529 167–171. 10.1038/nature16489 26700811

[B17] Drummond C. S. Eastwood R. J. Miotto S. T. Hughes C. E. (2012). Multiple continental radiations and correlates of diversification in *Lupinus* (Leguminosae): testing for key innovation with incomplete taxon sampling. *Syst. Biol.* 61 443–460. 10.1093/sysbio/syr126 22228799 PMC3329764

[B18] Edwards E. J. Donoghue M. J. (2013). Is it easy to move and easy to evolve? Evolutionary accessibility and adaptation. *J. Exp. Bot.* 64 4047–4052. 10.1093/jxb/ert220 23913955

[B19] Evans K. M. Waltz B. T. Tagliacollo V. A. Sidlauskas B. L. Albert J. S. (2017). Fluctuations in evolutionary integration allow for big brains and disparate faces. *Sci. Rep.* 7:40431. 10.1038/srep40431 28091543 PMC5238424

[B20] Evans M. E. K. Hearn D. Hahn W. Spangle J. Venable D. L. (2005). Climate and life-history evolution in evening primroses (Oenothera, Onagraceae): a phylogenetic comparative analysis. *Evolution* 59 1914–1927. 10.1111/j.0014-3820.2005.tb01061.x 16261729

[B21] Flexas J. Ribas-Carbó M. Diaz-Espejo A. Galmés J. Medrano H. (2008). Mesophyll conductance to CO_2_: current knowledge and future prospects. *Plant Cell Environ* 31 602–621. 10.1111/j.1365-3040.2007.01757.x 17996013

[B22] Friedman J. Rubin M. J. (2015). All in good time: understanding annual and perennial strategies in plants. *Am. J. Bot.* 102 497–499. 10.3732/ajb.1500062 25878083

[B23] Goswami A. Smaers J. B. Soligo C. Polly P. D. (2014). The macroevolutionary consequences of phenotypic integration: from development to deep time. *Philos. Trans. R. Soc. B* 369 1–15. 10.1098/rstb.2013.0254 25002699 PMC4084539

[B24] Gruenstaeudl M. Urtubey E. Jansen R. K. Samuel R. Barfuss M. H. J. Stuessy T. F. (2009). Phylogeny of Barnadesioideae (Asteraceae) inferred from DNA sequence data and morphology. *Mol. Phylogenet. Evol.* 51 572–587. 10.1016/j.ympev.2009.01.023 19264147

[B25] Harmon L. J. Weir J. T. Brock C. D. Glor R. E. Challenger W. (2008). GEIGER: investigating evolutionary radiations. *Bioinformatics* 24 129–131. 10.1093/bioinformatics/btm538 18006550

[B26] Harvey P. H. Pagel M. D. (1991). *The Comparative Method in Evolutionary Biology.* Oxford: Oxford University Press.

[B27] Heibl C. (2011). *PHYLOCLIM: Integrating Phylogenetics and Climatic Niche Modelling. R Package Version 0.9–4.* Available online at: https://cran.r-project.org/web/packages/phyloclim/index.html (accessed April 1, 2019).

[B28] Heibl C. Renner S. (2012). Distribution models and a dated phylogeny for Chilean *Oxalis* species reveal occupation of new habitats by different lineages, not rapid adaptive radiation. *Syst. Biol.* 61 823–834. 10.1093/sysbio/sys034 22357726

[B29] Hershkovitz M. A. Arroyo M. T. Bell C. Hinojosa L. F. (2006). Phylogeny of Chaetanthera (Asteraceae: Mutisieae) reveals both ancient and recent origins of the high elevation lineages. *Mol. Phylogenet. Evol.* 41 594–605. 10.1016/j.ympev.2006.05.003 16854602

[B30] Hijmans R. J. Cameron S. E. Parra J. L. Jones P. G. Jarvis A. (2005). Very high resolution interpolated climate surfaces for global land areas. *Int. J. Climatol.* 25 1965–1978. 10.1002/joc.1276

[B31] Hinojosa L. F. (2005). Climatic and vegetational changes inferred from Cenozoic Southern South America paleoflora. *Rev. Geol. Chile* 32 95–115.

[B32] Hinojosa L. F. Villagran C. (1997). History of the southern South American forests. 1. Paleobotanical, geological and climatical background on the Tertiary of southern South America. *Rev. Chil. Hist. Nat.* 70 225–239.

[B33] Houle D. Bolstad G. H. van der Linde K. Hansen T. F. (2017). Mutation predicts 40 million years of fly wing evolution. *Nature* 548 447–450. 10.1038/nature23473 28792935

[B34] Hughes C. E. Atchison G. W. (2015). The ubiquity of alpine plant radiations: from the Andes to the Hengduan Mountains. *New Phytol.* 2 275–282. 10.1111/nph.13230 25605002

[B35] Jara-Arancio P. Vidal P. Panero J. P. Marticorena A. Arroyo M. T. K. (2017). Phylogenetic reconstruction of the South American genus *Leucheria* Lag. (Asteraceae, Nassauvieae) based on nuclear and chloroplast DNA sequences. *Plant Syst. Evol.* 303 221–232. 10.1007/s00606-016-1366-7

[B36] Jímenez-Lobato V. Escudero M. Días-Linfante Z. Camacho C. A. de Castro A. Mansion G. (2019). Evolution of reproductive traits and selfing syndrome in the sub-endemic mediterranean genus *Centaurium* Hill. *Bot. J. Linn. Soc.* 191 216–235. 10.1093/botlinnean/boz036

[B37] Kidner C. Umbreen S. (2010). Why is leaf shape so variable? *Int. J. Plant Dev. Biol.* 4 64–75. 10.1093/aob/mcq261 21199835 PMC3043936

[B38] Klingenberg C. P. (2008). Morphological integration and developmental modularity. *Annu. Rev. Ecol. Evol.* 39 115–132. 10.1146/annurev.ecolsys.37.091305.110054

[B39] Klingenberg C. P. (2014). Studying morphological integration and modularity at multiple levels: concepts and analysis. *Philos. Trans. R. Soc. B.* 369:20130249. 10.1098/rstb.2013.0249 25002695 PMC4084535

[B40] Knight C. A. Ackerly D. D. (2003). Evolution and plasticity of photosynthetic thermal tolerance, specific leaf area and leaf size: congeneric species from desert and coastal environments. *New Phytol.* 160 337–347. 10.1046/j.1469-8137.2003.00880.x 33832168

[B41] Kooyers N. J. (2015). The evolution of drought escape and avoidance in natural herbaceous populations. *Plant Sci.* 234 155–162. 10.1016/j.plantsci.2015.02.012 25804818

[B42] Korner C. (2003). *Alpine Plant Life: Functional Plant Ecology of High Mountain Ecosystems.* Berlin: Springer.

[B43] Korner C. (2007). The use of “altitude” in ecological research. *Trends Ecol. Evol.* 22 569–574. 10.1016/j.tree.2007.09.006 17988759

[B44] Kudo G. (1991). Effects of snow-free period on the phenology of alpine plants inhabiting snow patches. *Arct. Alp. Res.* 23 436–443. 10.2307/1551685

[B45] Lande R. (1976). Natural selection and random genetic drift in phenotypic evolution. *Evolution* 30 314–334. 10.1111/j.1558-5646.1976.tb00911.x 28563044

[B46] Larouche O. Zelditch M. L. Cloutier R. (2018). Modularity promotes morphological divergence in ray-finned fishes. *Sci. Rep.* 8:7278. 10.1038/s41598-018-25715-y 29740131 PMC5940925

[B47] Lewontin R. C. (1978). Adaptation. *Sci. Am.* 239 212–231. 10.1038/scientificamerican0978-212 705323

[B48] Losos J. B. (2008). Phylogenetic niche conservatism, phylogenetic signal and the relationship between phylogenetic relatedness and ecological similarity among species. *Ecol. Lett.* 11 995–1003. 10.1111/j.1461-0248.2008.01229.x 18673385

[B49] Luebert F. (2011). Hacia una fitogeografía histórica del Desierto de Atacama. *Rev. Geogr. Norte Gd.* 50 105–133. 10.4067/S0718-34022011000300007 27315006

[B50] Luebert F. Weigend M. (2014). Phylogenetic insights into Andean plant diversification. *Front. Ecol. Evol.* 2:27 10.3389/fevo.2014.00027

[B51] Mason C. M. Donovan L. A. (2015). Evolution of the leaf economics spectrum in herbs: evidence from environmental divergences in leaf physiology across *Helianthus* (Asteraceae). *Evolution* 69 2705–2721. 10.1111/evo.12768 26339995

[B52] McGlothlin J. W. Kobiela M. E. Wright H. V. Mahler D. L. Kolbe J. J. Losso J. B. (2018). Adaptive radiation along a deeply conserved genetic line of least resistance in *Anolis* lizards. *Evol. Lett.* 2 310–322. 10.1002/evl3.72 30283684 PMC6121822

[B53] McGuigan K. Chenoweth S. F. Blows M. W. (2005). Phenotypic divergence along lines of genetic variance. *Am. Nat.* 165 32–43. 10.1086/426600 15729638

[B54] Nicolas A. N. Plunkett G. M. (2012). Untangling generic limits in *Azorella*, *Laretia*, and *Mulinum* (Apiaceae: Azorelloideae): insights from phylogenetics and biogeography. *Taxon* 61 826–840. 10.1002/tax.614008

[B55] Ogburn R. M. Edwards E. J. (2015). Life history lability underlies rapid climate niche evolution in the angiosperm clade Montiaceae. *Mol. Phylogenet. Evol.* 92 181–192. 10.1016/j.ympev.2015.06.006 26143714

[B56] Orme D. (2013). *The Caper Package: Comparative Analysis of Phylogenetics and Evolution in R. R package version 5.* Available online at: http://cran.r-project.org/package=caper (accessed November 1, 2019).

[B57] Pagel M. (1999). Inferring the historical patterns of biological evolution. *Nature* 401 877–884. 10.1038/44766 10553904

[B58] Paradis E. Claude J. Strimmer K. (2004). APE: analyses of phylogenetics and evolution in R language. *Bioinformatics* 20 289–290. 10.1093/bioinformatics/btg412 14734327

[B59] Phillips S. J. Anderson R. P. Schapired R. E. (2006). Maximum entropy modeling of species geographic distributions. *Ecol. Model.* 190 231–259. 10.1016/j.ecolmodel.2005.03.026

[B60] Pigliucci M. (2003). Phenotypic integation: studying the ecology and evolution of complex phenotypes. *Ecol. Lett.* 6 265–272. 10.1046/j.1461-0248.2003.00428.x

[B61] Poorter H. Niinemets U. Poorter L. Wright I. J. Villar R. (2009). Causes and consequences of variation in leaf mass per area (LMA): a meta-analysis. *New Phytol.* 182 565–588. 10.1111/j.1469-8137.2009.02830.x 19434804

[B62] Read Q. D. Moorhead L. C. Swenson N. G. Bailey J. K. Sanders N. J. (2014). Convergent effects of elevation on functional leaf traits within and among species. *Funct. Ecol.* 28 37–45. 10.1111/1365-2435.12162

[B63] Reich P. B. (2014). The world-wide ‘fast–slow’ plant economics spectrum: a traits manifesto. *J. Ecol.* 102 275–301. 10.1111/1365-2745.12211

[B64] Reich P. B. Walters M. B. Ellsworth D. S. (1997). From tropics to tundra: global convergence in plant functioning. *Proc. Natl. Acad. Sci. U.S.A.* 94 13730–13734. 10.1073/pnas.94.25.13730 9391094 PMC28374

[B65] Revell L. J. (2009). Size-correction and principal components for interspecific comparative studies. *Evolution* 63 3258–3268. 10.1111/j.1558-5646.2009.00804.x 19663993

[B66] Revell L. J. (2012). Phytools: an R package for phylogenetic comparative biology (and other things). *Methods Ecol. Evol.* 3 217–223. 10.1111/j.2041-210X.2011.00169.x

[B67] Revell L. J. Harmon L. J. Collar D. C. (2008). Phylogenetic signal, evolutionary process, and rate. *Syst. Biol.* 57 591–601. 10.1080/10635150802302427 18709597

[B68] Sack L. Hoolbrook N. M. (2006). Leaf hydraulics. 57 361–381. 10.1146/annurev.arplant.56.032604.144141 16669766

[B69] Schluter D. (1996). Adaptive radiation along genetic lines of least resistance. *Evolution* 50 1766–1774. 10.1111/j.1558-5646.1996.tb03563.x 28565589

[B70] Schluter D. (2000). *The Ecology of Adaptive Radiation.* Oxford: Oxford University Press.

[B71] Schneider C. A. Rasband W. S. Eliceiri K. W. (2012). NIH Image to ImageJ: 25 years of image analysis. *Nat. Methods* 9 671–675. 10.1038/nmeth.2089 22930834 PMC5554542

[B72] Simpson B. B. (1983). An historical phytogeography of the high Andean flora. *Rev. Chil. Hist. Nat.* 56 109–122.

[B73] Snell R. Aarssen L. W. (2005). Life history traits in selfing versus outcrossing annuals: exploring the “time-limitation” hypothesis for the fitness benefit of self-pollination. *BMC Ecol.* 5:2. 10.1186/1472-6785-5-2 15707481 PMC553978

[B74] Stebbins C. L. Jr. (1950). *Variation and Evolution in Plants.* New York, NY: Columbia University Press.

[B75] Tjoelker M. G. Craine J. M. Wedin D. Reich P. B. Tilman D. (2005). Linking leaf and root trait syndromes among grassland and savannah species. *New Phytol.* 167 493–508. 10.1111/j.1469-8137.2005.01428.x 15998401

[B76] Vile D. Garnier E. Shipley B. Laurent G. Navas M. L. Roumet C. (2005). Specific leaf area and dry matter content estimate thickness in laminar leaves. *Ann. Bot.* 96 1129–1136. 10.1093/aob/mci264 16159941 PMC4247101

[B77] Vogel S. (2009). Leaves in the lowest and highest winds: Temperature, force and shape: Tansley Review. *New Phytol.* 183 13–26. 10.1111/j.1469-8137.2009.02854.x 19413689

[B78] Wagner G. P. Altenberg L. (1996). Perspective: complex adaptations and the evolution of evolvability. *Evolution* 967–976. 10.1111/j.1558-5646.1996.tb02339.x 28565291

[B79] Wagner G. P. Pavlicev M. Cheverud J. M. (2007). The road to modularity. *Nat. Rev. Genet.* 8 921–931. 10.1038/nrg2267 18007649

[B80] Wiens J. J. Ackerly D. D. Allen A. P. Anacker B. L. Buckley L. B. Cornell H. V. (2010). Niche conservatism as an emerging principle in ecology and conservation biology. *Ecol. Lett.* 13 1310–1324. 10.1111/j.1461-0248.2010.01515.x 20649638

[B81] Wright I. J. Reich P. B. Westoby M. (2004). The worldwide leaf economics spectrum. *Nature* 428 821–827. 10.1038/nature02403 15103368

[B82] Yang Z. Kumar S. Nei M. (1995). A new method of inference of ancestral nucleotide and amino acid sequences. *Genetics* 141 1641–1650. 8601501 10.1093/genetics/141.4.1641 PMC1206894

[B83] Zanne A. E. Tank D. C. Cornwell W. K. Eastman J. M. Smith S. A. FitzJohn R. G. (2014). Three keys to the radiation of angiosperms into freezing environments. *Nature* 506 89–92. 10.1038/nature12872 24362564

