# Supplementary material for: Divergence in Plant Traits and Increased Modularity Underlie Repeated Transitions Between Low and High Elevations in the Andean Genus Leucheria
Source: Front Plant Sci. 2020 Jun 4;11:714. doi: 10.3389/fpls.2020.00714 (PMC7287153; doi:10.3389/fpls.2020.00714)
Supplement: Supplementary file 2 [file Table_2.DOCX]

**Supplementary Material**

Appendix 2. Information on location, climatic niche variables and plant traits of the 34 studies *Leucheria* species. Habitat: WA, warm arid-semiarid shrublands at low to mid altitudes of Coastal Desert and Mediterranean region; HF, humid forest; CS, cold steppe at high-elevations (above tree line) or latitudes in Patagonian. Climatic niche variables derived from Predicted Niche Occupancy Profiles: wMAT, weighted mean annual temperature; wMAP, weighted mean annual precipitation; p25, 25^th^ percentile, p75, 75^th^ percentile. Plant Traits: PH, plant height; LMA, leaf mass per area; LD, leaf dissection, LT; leaf thickness; LA; leaf area; AR, leaf aspect ratio; RSR, root/shoot ratio. Values represent the mean ± standard error of plant traits of 10 individuals per species.

| CODE | Species | Elev. | | Lat | | Lon | | Habitat | | wMAT (p25-p75) | | wMAP (p25-p75) | | PH | LA | | LMA | | LT | | AR | | LD | RSR | |  |  |  |
| --- | --- | --- | --- | --- | --- | --- | --- | --- | --- | --- | --- | --- | --- | --- | --- | --- | --- | --- | --- | --- | --- | --- | --- | --- | --- | --- | --- | --- |
|  |  | m | | °S | | °W | |  | | °C | | mm | | m | cm^2^ | | gm^-2^ | | mm | |  | |  |  | |  |  |  |
| ACHI | *achillaeifolia* | 647 | | -50.8 | | -72.5 | | CS | | 7.42 (5.8-8.8) | | 494 (236-664) | | 0.21±0.07 | 1.96±0.24 | | 76.5±4.0 | | 0.23±0.02 | | 4.34±0.58 | | 2.96±0.09 | 1.14±0.22 | |  |  |  |
| AMO | *amoena* | 1210 | | -35.1 | | -70.6 | | WA | | 8.18 (3.8-11.9) | | 765 (281-930) | | 0.98±0.17 | 17.84±1.86 | | 68.9±2.5 | | 0.33±0.01 | | 3.99±0.13 | | 2.88±0.2 | 0.94±0.18 | |  |  |  |
| API | *apiifolia* | 2896 | | -33.3 | | -70.3 | | CS | | 3.78 (-1.4-8.7) | | 751 (434-930) | | 0.53±0.09 | 22.2±3.07 | | 53.2±2.6 | | 0.20±0.00 | | 4.24±0.34 | | 2.70±0.41 | 0.61±0.07 | |  |  |  |
| BRI | *bridgesii* | 2170 | | -34.1 | | -70 | | CS | | 3.95 (-0.9-7.9) | | 441 (297-553) | | 0.43±0.08 | 2.07±0.13 | | 91.6±2.6 | | 0.31±0.01 | | 5.44±0.19 | | 3.09±0.06 | 1.61±0.27 | |  |  |  |
| CAN | *candidissima* | 2505 | | -35.2 | | -70.5 | | CS | | 5.75 (3.4-8.1) | | 482 (202-673) | | 0.08±0.01 | 0.84±0.06 | | 150.7±4.8 | | 0.32±0.00 | | 5.47±0.25 | | 3.50±0.42 | 2.11±0.3 | |  |  |  |
| CER | *cerberoana* | 30 | | -28.1 | | -71.2 | | WA | | 14.04 (12.8-15.9) | | 229 (56-259) | | 0.14±0.04 | 3.71±0.14 | | 46.9±1.9 | | 0.19±0.00 | | 2.05±0.16 | | 2.62±0.39 | 0.03±0.01 | |  |  |  |
| COE | *coerulescens* | 1384 | | -35.5 | | -71 | | HF | | 7.21 (5.6-9.0) | | 1209 (712-1791) | | 0.81±0.13 | 8.71±0.87 | | 42.8±1.58 | | 0.17±0.00 | | 3.84±0.24 | | 2.56±0.03 | 0.22±0.04 | |  |  |  |
| CON | *congesta* | 2420 | | -33.4 | | -70.3 | | CS | | 4.17 (0.0-7.9) | | 427 (202-581) | | 0.97±0.12 | 3.42±0.12 | | 66.4±1.3 | | 0.25±0.01 | | 4.05±0.22 | | 2.85±0.23 | 2.82±0.54 | |  |  |  |
| CUM | *cumingii* | 700 | | -25.4 | | -70.5 | | WA | | 15.64 (14.4-17.2) | | 92 (9-16) | | 0.23±0.04 | 2.98±0.30 | | 51.6±2.1 | | 0.19±0.01 | | 3.26±0.13 | | 2.68±0.38 | 0.07±0.01 | |  |  |  |
| FLO | *floribunda* | 1421 | | -33.8 | | -70.2 | | WA | | 4.88 (2.3-7.4) | | 471 (211-683) | | 1.40±0.16 | 8.92±1.18 | | 87.4±2.0 | | 0.30±0.01 | | 4.31±0.12 | | 3.06±0.31 | 0.56±0.1 | |  |  |  |
| GAR | *garciana* | 2372 | | -35.2 | | -70.5 | | CS | | 5.00 (-0.7-9.4) | | 711 (406-831) | | 0.37±0.03 | 4.35±0.38 | | 82.2±1.6 | | 0.29±0.01 | | 6.92±0.36 | | 3.01±0.27 | 1.90±0.19 | |  |  |  |
| GAY | *gayana* | 2386 | | -33.4 | | -70.3 | | CS | | 6.63 (2.5-10.1) | | 539 (200-750) | | 0.47±0.07 | 8.95±0.65 | | 71.9±3.2 | | 0.20±0.00 | | 3.69±0.15 | | 2.91±0.15 | 1.92±0.21 | |  |  |  |
| GIL | *gilliesi* | 2260 | | -36 | | -70.6 | | CS | | 7.60 (3.3-12.1) | | 690 (246-813) | | 0.33±0.05 | 8.70±0.86 | | 77.9±3.3 | | 0.30±0.01 | | 5.55±0.38 | | 2.97±0.30 | 0.90±0.15 | |  |  |  |
| GLA | *glacialis* | 2406 | | -35.2 | | -70.5 | | CS | | 7.09 (5.4-8.4) | | 885 (658-1076) | | 0.56±0.08 | 8.63±0.47 | | 86.8±2.4 | | 0.27±0.00 | | 7.56±0.57 | | 3.05±0.21 | 1.46±0.22 | |  |  |  |
| GLD | *glandulosa* | 724 | | -34.8 | | -70.7 | | WA | | 8.42 (3.1-14.3) | | 532 (152-644) | | 0.42±0.04 | 2.62±0.36 | | 46.0±2.1 | | 0.12±0.01 | | 3.59±0.20 | | 2.60±0.10 | 0.08±0.01 | |  |  |  |
| HAH | *hahnnii* | 700 | | -51.1 | | -72.8 | | CS | | 5.08 (3.9-6.2) | | 502 (290-554) | | 0.22±002 | 0.39±0.02 | | 111.4±2.7 | | 0.31±0.01 | | 4.24±0.25 | | 3.25±0.21 | 1.42±0.17 | |  |  |  |
| HIE | *hieracioides* | 1481 | | -33.4 | | -70.5 | | WA | | 5.66 (0.7-10.2) | | 576 (363-767) | | 0.95±0.13 | 11.2±0.91 | | 42.7±1.7 | | 0.23±0.00 | | 3.39±0.12 | | 2.56±0.13 | 0.05±0.01 | |  |  |  |
| INT | *integrifolia* | 1966 | | -35.1 | | -70.5 | | CS | | 4.58 (4.4-7.2) | | 690 (353-788) | | 0.07±0.02 | 8.03±0.37 | | 65.2±1.9 | | 0.27±0.00 | | 5.82±0.23 | | 2.84±0.17 | 0.84±0.17 | |  |  |  |
| LEO | *leontopodioides* | 1065 | | -50.8 | | -72.5 | | CS | | 6.16 (-0.9-9.3) | | 457 (300-547) | | 0.58±0.12 | 0.46±0.02 | | 230.1±11.1 | | 0.42±0.01 | | 1.73±0.05 | | 3.89±0.31 | 3.13±0.56 | |  |  |  |
| LIT | *lithospermifolia* | 1210 | | -35.1 | | -70.6 | | WA | | 6.96 (5.5-8.5) | | 975 (797-1107) | | 1.13±0.26 | 8.57±0.60 | | 92.9±3.6 | | 0.30±0.00 | | 5.74±0.32 | | 3.10±0.28 | 1.61±0.32 | |  |  |  |
| MIL | *millefolium* | 2523 | | -35.2 | | -70.5 | | CS | | 6.71 (5.1-8.3) | | 455 (191-651) | | 0.17±0.03 | 1.61±0.06 | | 192.4±7.5 | | 0.42±0.01 | | 2.70±0.13 | | 3.72±0.26 | 2.15±0.37 | |  |  |  |
| MUL | *multiflora* | 90 | | -31.8 | | -71.5 | | WA | | 14.60 (9.7-18.5) | | 476 (43-705) | | 0.41±0.08 | 3.29±0.26 | | 83.3±3.9 | | 0.20±0.01 | | 3.15±0.22 | | 3.02±0.06 | 1.50±0.26 | |  |  |  |
| OLI | *oligocephala* | 900 | | -33.7 | | -70.5 | | WA | | 9.90 (4.8-14.8) | | 392 (123-592) | | 0.38±0.06 | 2.91±0.21 | | 37.3±1.0 | | 0.16±0.00 | | 3.79±0.18 | | 2.47±0.27 | 0.08±0.02 | |  |  |  |
| PAP | *papilosa* | 210 | | -52.1 | | -71.4 | | CS | | 6.48 (4.3-8.4) | | 739 (221-1025) | | 0.21±0.03 | 1.13±0.15 | | 76.3±2.35 | | 0.22±0.00 | | 3.01±0.12 | | 2.96±0.18 | 1.49±0.28 | |  |  |  |
| POL | *polyclados* | 3153 | | -27.9 | | -69.4 | | CS | | 4.37 (0.7-6.9) | | 81 (38-116) | | 0.29±0.04 | 2.13±0.20 | | 109.1±2.10 | | 0.29±0.00 | | 3.16±0.20 | | 3.23±0.42 | 0.40±0.05 | |  |  |  |
| PUR | *purpurea* | 647 | | -50.8 | | -72.5 | | CS | | 5.46 (4.4-6.3) | | 477 (303-575) | | 0.13±0.02 | 0.59±0.05 | | 91.5±2.5 | | 0.30±0.01 | | 2.94±0.11 | | 3.09±0.31 | 1.22±0.13 | |  |  |  |
| ROS | *rosea* | 1437 | | -33.8 | | -70.2 | | CS | | 5.45 (1.0-9.1) | | 529 (389-656) | | 0.92±0.11 | 5.37±0.42 | | 89.9±2.0 | | 0.23±0.00 | | 5.73±0.31 | | 3.08±0.09 | 0.55±0.11 | |  |  |  |
| RUN | *runcinata* | 2740 | | -33.4 | | -70.3 | | CS | | 4.53 (-0.3-8.5) | | 378 (201-476) | | 0.97±0.15 | 26.9±4.01 | | 67.5±3.1 | | 0.23±0.00 | | 4.25±0.32 | | 2.87±0.29 | 0.46±0.05 | |  |  |  |
| SAL | *salina* | 3595 | | -33.3 | | -70.3 | | CS | | 1.98 (-0.2-6.0) | | 366 (180-470) | | 0.21±0.03 | 4.59±0.08 | | 146.6±2.8 | | 0.31±0.01 | | 4.12±0.24 | | 3.48±0.11 | 0.82±0.16 | |  |  |  |
| SCR | *scrobiculata* | 3412 | | -34.2 | | -69.8 | | CS | | 3.53 (-1.4-8.0) | | 430 (181-548) | | 0.07±0.04 | 0.43±0.03 | | 212.8±9.8 | | 0.40±0.02 | | 4.05±0.25 | | 3.82±0.31 | 0.99±0.11 | |  |  |  |
| SEN | *senecioides* | 25 | | -34.1 | | -72 | | WA | | 12.82 (11.5-14.6) | | 801 (495-1037) | | 0.26±0.06 | 0.89±0.07 | | 69.7±2.1 | | 0.24±0.00 | | 4.32±0.26 | | 2.89±0.32 | 0.08±0.01 | |  |  |  |
| TEN | *tenuis* | 1410 | | -33.4 | | -70.5 | | WA | | 8.25 (3.3-13.6) | | 475 (294-591) | | 0.36±0.02 | 0.19±0.02 | | 26.3±2.0 | | 0.07±0.00 | | 7.16±0.37 | | 2.26±0.21 | 0.12±0.02 | |  |  |  |
| THE | *thermarum* | 1284 | | -38.2 | | -71.7 | | HF | | 7.89 (5.9-9.5) | | 1191 (880-1463) | | 0.76±0.09 | 9.78±0.70 | | 32.1±1.8 | | 0.12±0.01 | | 2.24±0.16 | | 2.38±0.14 | 0.47±0.08 | |  |  |  |
| TOM | *tomentosa* | 2 | | -31.8 | | -71.5 | | WA | | 13.70 (12.1-15.7) | | 446 (242-536) | | 0.27±0.04 | 0.87±0.05 | | 76.2±2.45 | | 0.24±0.00 | | 3.68±0.13 | | 2.95±0.27 | 0.15±0.02 | |  |  |  |
|  |  | |  | |  | |  | |  | |  | |  | | |  | |  | |  | |  | | |  | |  |  |
|  |  | |  | |  | |  | |  | |  | |  | | |  | |  | |  | |  | | |  | |  |  |
|  |  | |  | |  | |  | |  | |  | |  | | |  | |  | |  | |  | | |  | |  |  |
|  |  | |  | |  | |  | |  | |  | |  | | |  | |  | |  | |  | | |  | |  |  |
|  |  | |  | |  | |  | |  | |  | |  | | |  | |  | |  | |  | | |  | |  |  |
|  |  | |  | |  | |  | |  | |  | |  | | |  | |  | |  | |  | | |  | |  |  |
|  |  | |  | |  | |  | |  | |  | |  | | |  | |  | |  | |  | | |  | |  |  |
|  |  | |  | |  | |  | |  | |  | |  | | |  | |  | |  | |  | | |  | |  |  |
|  |  | |  | |  | |  | |  | |  | |  | | |  | |  | |  | |  | | |  | |  |  |
|  |  | |  | |  | |  | |  | |  | |  | | |  | |  | |  | |  | | |  | |  |  |
|  |  | |  | |  | |  | |  | |  | |  | | |  | |  | |  | |  | | |  | |  |  |
|  |  | |  | |  | |  | |  | |  | |  | | |  | |  | |  | |  | | |  | |  |  |
|  |  | |  | |  | |  | |  | |  | |  | | |  | |  | |  | |  | | |  | |  |  |
|  |  | |  | |  | |  | |  | |  | |  | | |  | |  | |  | |  | | |  | |  |  |
|  |  | |  | |  | |  | |  | |  | |  | | |  | |  | |  | |  | | |  | |  |  |
|  |  | |  | |  | |  | |  | |  | |  | | |  | |  | |  | |  | | |  | |  |  |
|  |  | |  | |  | |  | |  | |  | |  | | |  | |  | |  | |  | | |  | |  |  |
|  |  | |  | |  | |  | |  | |  | |  | | |  | |  | |  | |  | | |  | |  |  |
|  |  | |  | |  | |  | |  | |  | |  | | |  | |  | |  | |  | | |  | |  |  |
|  |  | |  | |  | |  | |  | |  | |  | | |  | |  | |  | |  | | |  | |  |  |
|  |  | |  | |  | |  | |  | |  | |  | | |  | |  | |  | |  | | |  | |  |  |
|  |  | |  | |  | |  | |  | |  | |  | | |  | |  | |  | |  | | |  | |  |  |
|  |  | |  | |  | |  | |  | |  | |  | | |  | |  | |  | |  | | |  | |  |  |
|  |  | |  | |  | |  | |  | |  | |  | | |  | |  | |  | |  | | |  | |  |  |
|  |  | |  | |  | |  | |  | |  | |  | | |  | |  | |  | |  | | |  | |  |  |
|  |  | |  | |  | |  | |  | |  | |  | | |  | |  | |  | |  | | |  | |  |  |
|  |  | |  | |  | |  | |  | |  | |  | | |  | |  | |  | |  | | |  | |  |  |
|  |  | |  | |  | |  | |  | |  | |  | | |  | |  | |  | |  | | |  | |  |  |
|  |  | |  | |  | |  | |  | |  | |  | | |  | |  | |  | |  | | |  | |  |  |
|  |  | |  | |  | |  | |  | |  | |  | | |  | |  | |  | |  | | |  | |  |  |
|  |  | |  | |  | |  | |  | |  | |  | | |  | |  | |  | |  | | |  | |  |  |
|  |  | |  | |  | |  | |  | |  | |  | | |  | |  | |  | |  | | |  | |  |  |
|  |  | |  | |  | |  | |  | |  | |  | | |  | |  | |  | |  | | |  | |  |  |
|  |  | |  | |  | |  | |  | |  | |  | | |  | |  | |  | |  | | |  | |  |  |
|  |  | |  | |  | |  | |  | |  | |  | | |  | |  | |  | |  | | |  | |  |  |
|  |  | |  | |  | |  | |  | |  | |  | | |  | |  | |  | |  | | |  | |  |  |

|  |  |  |  |  |  |  |  |  |  |  |  |  |  |  |
| --- | --- | --- | --- | --- | --- | --- | --- | --- | --- | --- | --- | --- | --- | --- |
|  |  |  |  |  |  |  |  |  |  |  |  |  |  |  |
|  |  |  |  |  |  |  |  |  |  |  |  |  |  |  |
|  |  |  |  |  |  |  |  |  |  |  |  |  |  |  |
|  |  |  |  |  |  |  |  |  |  |  |  |  |  |  |
|  |  |  |  |  |  |  |  |  |  |  |  |  |  |  |
|  |  |  |  |  |  |  |  |  |  |  |  |  |  |  |
|  |  |  |  |  |  |  |  |  |  |  |  |  |  |  |
|  |  |  |  |  |  |  |  |  |  |  |  |  |  |  |
|  |  |  |  |  |  |  |  |  |  |  |  |  |  |  |
|  |  |  |  |  |  |  |  |  |  |  |  |  |  |  |
|  |  |  |  |  |  |  |  |  |  |  |  |  |  |  |
|  |  |  |  |  |  |  |  |  |  |  |  |  |  |  |
|  |  |  |  |  |  |  |  |  |  |  |  |  |  |  |
|  |  |  |  |  |  |  |  |  |  |  |  |  |  |  |
|  |  |  |  |  |  |  |  |  |  |  |  |  |  |  |
|  |  |  |  |  |  |  |  |  |  |  |  |  |  |  |
|  |  |  |  |  |  |  |  |  |  |  |  |  |  |  |
|  |  |  |  |  |  |  |  |  |  |  |  |  |  |  |
|  |  |  |  |  |  |  |  |  |  |  |  |  |  |  |
|  |  |  |  |  |  |  |  |  |  |  |  |  |  |  |
|  |  |  |  |  |  |  |  |  |  |  |  |  |  |  |
|  |  |  |  |  |  |  |  |  |  |  |  |  |  |  |
|  |  |  |  |  |  |  |  |  |  |  |  |  |  |  |
|  |  |  |  |  |  |  |  |  |  |  |  |  |  |  |
|  |  |  |  |  |  |  |  |  |  |  |  |  |  |  |
|  |  |  |  |  |  |  |  |  |  |  |  |  |  |  |
|  |  |  |  |  |  |  |  |  |  |  |  |  |  |  |
|  |  |  |  |  |  |  |  |  |  |  |  |  |  |  |
|  |  |  |  |  |  |  |  |  |  |  |  |  |  |  |
|  |  |  |  |  |  |  |  |  |  |  |  |  |  |  |
|  |  |  |  |  |  |  |  |  |  |  |  |  |  |  |
|  |  |  |  |  |  |  |  |  |  |  |  |  |  |  |
|  |  |  |  |  |  |  |  |  |  |  |  |  |  |  |
|  |  |  |  |  |  |  |  |  |  |  |  |  |  |  |
|  |  |  |  |  |  |  |  |  |  |  |  |  |  |  |
